# Modeling the interaction between tubuloglomerular feedback and myogenic mechanisms in the control of glomerular mechanics

**DOI:** 10.3389/fphys.2024.1410764

**Published:** 2024-06-20

**Authors:** Owen Richfield, Ricardo Cortez, L. Gabriel Navar

**Affiliations:** ^1^ Bioinnovation PhD Program, Tulane University, New Orleans, LA, United States; ^2^ Department of Mathematics, Tulane University, New Orleans, LA, United States; ^3^ Department of Physiology, Tulane School of Medicine, New Orleans, LA, United States

**Keywords:** glomerulus, mathematical modeling, renal autoregulation, tubuloglomerular feedback, myogenic

## Abstract

**Introduction:** Mechanical stresses and strains exerted on the glomerular cells have emerged as potentially influential factors in the progression of glomerular disease. Renal autoregulation, the feedback process by which the afferent arteriole changes in diameter in response to changes in blood pressure, is assumed to control glomerular mechanical stresses exerted on the glomerular capillaries. However, it is unclear how the two major mechanisms of renal autoregulation, the afferent arteriole myogenic mechanism and tubuloglomerular feedback (TGF), each contribute to the maintenance of glomerular mechanical homeostasis.

**Methods:** In this study, we made a mathematical model of renal autoregulation and combined this model with an anatomically accurate model of glomerular blood flow and filtration, developed previously by us. We parameterized the renal autoregulation model based on data from previous literature, and we found evidence for an increased myogenic mechanism sensitivity when TGF is operant, as has been reported previously. We examined the mechanical effects of each autoregulatory mechanism (the myogenic, TGF and modified myogenic) by simulating blood flow through the glomerular capillary network with and without each mechanism operant.

**Results:** Our model results indicate that the myogenic mechanism plays a central role in maintaining glomerular mechanical homeostasis, by providing the most protection to the glomerular capillaries. However, at higher perfusion pressures, the modulation of the myogenic mechanism sensitivity by TGF is crucial for the maintenance of glomerular mechanical homeostasis. Overall, a loss of renal autoregulation increases mechanical strain by up to twofold in the capillaries branching off the afferent arteriole. This further corroborates our previous simulation studies, that have identified glomerular capillaries nearest to the afferent arteriole as the most prone to mechanical injury in cases of disturbed glomerular hemodynamics.

**Discussion:** Renal autoregulation is a complex process by which multiple feedback mechanisms interact to control blood flow and filtration in the glomerulus. Importantly, our study indicates that another function of renal autoregulation is control of the mechanical stresses on the glomerular cells, which indicates that loss or inhibition of renal autoregulation may have a mechanical effect that may contribute to glomerular injury in diseases such as hypertension or diabetes. This study highlights the utility of mathematical models in integrating data from previous experimental studies, estimating variables that are difficult to measure experimentally (i.e. mechanical stresses in microvascular networks) and testing hypotheses that are historically difficult or impossible to measure.

## Introduction

Alterations in mechanical stresses on the glomerular capillaries have been implicated in the progression of glomerulopathy in numerous kidney diseases (Endlich and Endlich, 2012; Kriz and Lemley, 2015; Endlich et al., 2017; Kriz and Lemley, 2017; Srivastava et al., 2017), and the magnitude of the mechanical stresses in the glomerulus depends on the efficiency of autoregulatory control. In the renal microcirculation, the myogenic and tubuloglomerular feedback (TGF) mechanisms of renal autoregulation maintain glomerular blood flow and pressure at optimum levels (Navar et al., 2008). These mechanisms respond to different chemical and physical signals; the myogenic mechanism causes a fast constriction of the afferent arteriole in response to increases in afferent arteriole wall tension, whereas TGF causes slower constriction and is mediated by signals from the macula densa cells that sense increases in tubular fluid osmolality or sodium concentration. Both mechanisms converge at the level of the afferent arteriole smooth muscle cells (SMC), and it is unclear to what degree the TGF and myogenic mechanisms dynamically interact to control SMC tone (Cupples, 2007).

Numerous studies have supported the theory that TGF modulates the sensitivity of the myogenic mechanism (Walker et al., 2000; Walker, 2001; Scully et al., 2013; Mitrou et al., 2015; Scully et al., 2016; Scully et al., 2017); the afferent arteriole constricts faster and with greater magnitude when TGF is intact as opposed to when TGF is inoperant (Walker et al., 2000; Walker, 2001), but it is unclear if this is a result of the TGF mechanism acting additively with the myogenic mechanism, or whether the TGF mechanism directly influences the magnitude and/or kinetics of the myogenic response. Mathematical models of hemodynamic autoregulation allow for the interrogation of hypotheses regarding relative strengths of the myriad factors controlling SMC tone (Carlson and Secomb, 2005; Carlson et al., 2008; Sgouralis and Layton, 2014). We developed a model of renal autoregulation that combines our previously developed model of blood flow and filtration in an anatomically accurate rat glomerular capillary network (Richfield et al., 2020; Richfield et al., 2021) with models of the renal tubule and afferent arteriole to quantitatively characterize interactions between the myogenic and TGF mechanisms and their impact on glomerular mechanics.

The model presented here provides estimates of the mechanical stresses and strains in the glomerular capillaries under different autoregulatory conditions. By modifying the magnitude and kinetics of the autoregulatory mechanisms and their interactions, we estimate each mechanism’s impact on the magnitude of different glomerular mechanical stresses. While previous studies have used mathematical models to comprehensively investigate renal autoregulatory dynamics (Sgouralis and Layton, 2012; Edwards and Layton, 2014; Sgouralis and Layton, 2014; Sgouralis and Layton, 2015; Sgouralis et al., 2016; Ciocanel et al., 2018), no studies have quantitatively related these dynamics to the mechanical consequences experienced by the glomerular cells. Our goal in this study was to quantify the contribution of each autoregulatory mechanism and their interactions to glomerular mechanical homeostasis. Our results indicate that the TGF mechanism directly modulates the sensitivity of the myogenic mechanism, and that this interaction is required to maintain mechanical homeostasis of the glomerular cells when blood pressure is elevated. These findings corroborate previous studies of myogenic mechanism-TGF interaction, suggest mechanisms of glomerular injury in diseases such as hypertension and diabetes, and highlight the utility of mathematical models in probing questions in renal physiology.

## Mathematical model

**FIGURE 1:**
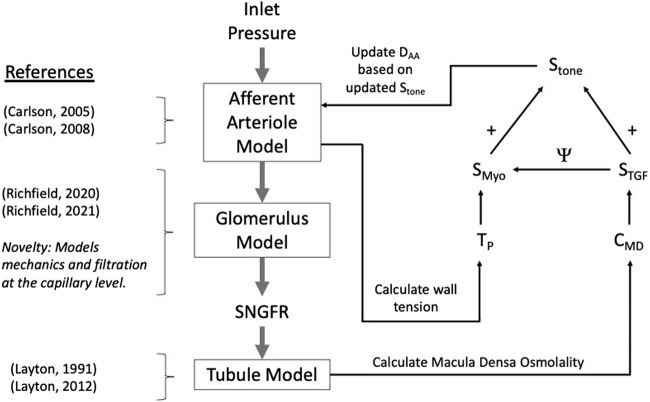
Autoregulation model schematic. Models of the glomerulus and tubule are used to estimate SNGFR and macula densa solute concentration, C_MD_. The afferent arteriole model calculates wall tension T_P_, and T_P_ and C_MD_ are used to calculate the myogenic and TGF tones, denoted S_Myo_ and S_TGF_, respectively. The interaction between S_Myo_ and S_TGF_ is denoted by Ψ in the diagram and will be referred to as such throughout this article. References are included to indicate the core references used to construct each model, with the novelty of the glomerulus sub-model accentuated.

We developed a model of renal autoregulation that portrays the interaction of the TGF and myogenic mechanisms in maintaining single nephron glomerular filtration rate (SNGFR). The model was composed of an afferent arteriole model, a glomerulus model and a tubule model run in series such that the output of each model was used as input for the next (Figure 1). The afferent arteriole model was derived from a model of cerebral autoregulation (Carlson et al., 2008) and the model was fit to data from previous studies that used the juxtamedullary nephron preparation to measure changes in afferent arteriole diameter and flow in response to changes in perfusion pressure (Takenaka et al., 1994; Walker et al., 2000). A glomerulus model previously developed by us (Richfield et al., 2020) was used to estimate magnitudes of SNGFR and mechanical stresses in the glomerular capillaries. To model solute exchange on the length of the tubule, we used a model of solute concentration along the relevant tubular segments (proximal tubule and the descending and ascending limbs of the Loop of Henle) to estimate macula densa solute concentration as a function of SNGFR (Layton et al., 1991). We briefly discuss each sub-model and describe the parameterization of the renal autoregulation model.

### Glomerulus model

To estimate the effect of alterations in afferent arteriole diameter on glomerular filtration and mechanics, we used a previously developed model of glomerular hemodynamics that models blood flow and filtration throughout an anatomically accurate glomerular capillary network (Richfield et al., 2020). We briefly describe the main equations of the model and discuss our methods for allowing for elastic deformation of the simulated glomerular capillaries, which we have modeled previously (Richfield et al., 2021). Incorporation of an anatomically accurate model of the glomerulus into our model of renal autoregulation constitutes a novel step forward in modeling renal autoregulation, as this model allows us to estimate filtration dynamics and mechanical stress at the glomerular capillary level. This has never been done in previous models of autoregulation.

The anatomical data used in this model were obtained via perfusion fixation and ultrathin sectioning in a previous study (Shea, 1979). The glomerular capillary network used in the model is composed of 320 capillary segments with known length and diameter, and 195 nodes at which the capillary segments bifurcate and/or coalesce. The glomerulus model uses conservation laws to calculate changes in plasma protein concentration and hematocrit as the plasma water is filtered along the length of the network. Thus, for a given systemic plasma protein concentration C_A_ and hematocrit H_t_, we calculate the concentration of plasma protein, C and erythrocyte volume in each capillary in the network. Erythrocyte volume is distributed at network nodes nonlinearly according to previously developed empirical models of blood phase separation in the microvasculature (Pries et al., 1996) and the hematocrit in each capillary segment nonlinearly affects blood viscosity according to previous experimental findings (Pries et al., 1994). Improving on previous models of blood flow and filtration in an anatomically accurate rat glomerular capillary network (Lambert et al., 1982; Remuzzi et al., 1992), our glomerulus model does not assume a linear pressure profile on the length of each capillary but instead takes the filtration of fluid into account in calculating the pressure profile p(x). For x = 0 to the capillary length, denoted L, and for R^f^ the resistance of the glomerular capillary wall to filtration, we obtain the second-order differential equation for the pressure profile:

$$\frac{d^{2}p}{dx^{2}}\left(x\right)-a^{2}p\left(x\right)=-a^{2}p_{BS},$$ (1)

Where p_BS_ denotes Bowman’s Space pressure, a^2^ = R/(R^f^ L^2^) for R the capillary resistance, which we calculate assuming Poiseuille flow. Glomerular model parameters are available in Table 3. The apparent viscosity μ in each capillary segment is nonlinearly dependent on plasma viscosity μ^pl^, hematocrit H_t_ and the capillary diameter D (Pries et al., 1994):

$$\mu =\mu^{pl}\lambda \left(D,H_{t}\right).$$ (2)

In contrast to our previous publications (Richfield et al., 2020; Richfield et al., 2021), λ is defined as in (Remuzzi et al., 1992). We obtain the filtered volume or “capillary segment glomerular filtration rate” (CSGFR) by integrating over the length of each glomerular capillary:

$$CSGFR=\frac{\int_{0}^{L}\left(p\left(x\right)-p_{BS}\right)dx}{R^{f}L},$$ (3)

and total SNGFR is taken to be the sum of the individual CSGFRs. The filtration resistance R^f^ is not fixed but is calculated iteratively as a function of plasma protein concentration and the pressure profile on the length of the capillary. According to the fundamental equations of glomerular filtration (Deen et al., 1972) the blood flow through each capillary changes as a function of the pressure profile p(x) on the length of the capillary and the colloid osmotic pressure Π(x) that opposes filtration according to the concentration of plasma protein within the capillary lumen:

$$\frac{dQ}{dx}=-k\pi D\left(p\left(x\right)-p_{BS}-\Pi \left(x\right)\right),$$ (4)

For k the hydraulic conductivity of the glomerular capillary wall, defined as the permeability of the wall to water, D the capillary diameter, and

$$\Pi \left(x\right)=2.1C\left(x\right)+0.16C^{2}\left(x\right)+0.009C^{3}\left(x\right).$$ (5)

(Papenfuss and Gross, 1978). Assuming Poiseuille flow,

$$Q\left(x\right)=-\frac{L}{R}\frac{dp}{dx}\left(x\right).$$ (6)

Taking the derivative with respect to x,

$$\frac{dQ}{dx}\left(x\right)=-\frac{L}{R}\frac{d^{2}p}{dx^{2}}\left(x\right)=a^{2}\left(p\left(x\right)-p_{BS}\right).$$ (7)

To enforce equality between Equations 4 and 7 we let

$$R^{f}=\frac{\int_{0}^{L}\left(p\left(x\right)-p_{BS}\right)dx}{k\pi DL\int_{0}^{L}\left(p\left(x\right)-p_{BS}-\Pi \left(x\right)\right)dx}.$$ (8)

This formulation allows for R^f^ to be iteratively updated for each capillary segment, as discussed in our previous work (Richfield et al., 2020).

To assess pressure boundary conditions for Equation (10), we calculate the pressure at each network node assuming conservation of blood flow, Q. Specifically, if we let J be the set of nodes j connected to node i, conservation of blood flow at node i is represented by

$$\sum_{j\in J}Q_{ij}=0,$$ (9)

Where Q_ij_ denotes the blood flow between nodes i and j through capillary ij. This relation defines a system of linear equations that can be used to calculate node pressures simultaneously, given pressure boundary conditions at the inlet and outlet, denoted P_a_ and P_e_, respectively. In model simulations, P_a_ and P_e_ are set equal to mean arterial pressure and peritubular capillary pressure, respectively.

In addition to predicting aspects of glomerular function, our glomerulus model estimates mechanical stresses in the individual glomerular capillaries. We calculate shear stress, τ assuming Poiseuille flow:

$$\tau =\frac{32\mu \frac{1}{L}\int_{0}^{L}Q\left(x\right)dx}{\pi D^{3}}.$$ (10)

Hoop stresses on each capillary segment, denoted by σ are calculated using the Young–Laplace equation:

$$\sigma =\frac{D\frac{1}{L}\left(\int_{0}^{L}\left(p\left(x\right)-p_{BS}\right)dx\right)}{2t_{GFB}},$$ (11)

For t_GFB_ the thickness of the glomerular filtration barrier. Further details of the glomerulus model algorithm and derivation are available in our previous work (Richfield et al., 2020).

In previous iterations of the model (Richfield et al., 2020), the afferent and efferent arterioles were represented as *fixed* resistors that were tuned to recapitulate rat glomerular hemodynamics in control and disease conditions (Zatz et al., 1986; Kasiske et al., 1988; Franco et al., 2011). In the current study, the afferent arteriole diameter changes as a function of perfusion pressure, which in turn affects pressure and filtration in the glomerulus. In our model formulation the afferent arteriole model determines the change in D_AA_ based on autoregulatory inputs and this diameter is translated to an afferent resistance R_AA_ in the glomerular model. The efferent arteriole length L_EA_ and diameter D_EA_ remain fixed and are used to maintain an adequate glomerular pressure in the control state (Sgouralis and Layton, 2014).

To calculate strain (stretch) of the glomerular capillary walls, we use a constitutive relation assuming that the glomerular filtration barrier is a neo-Hookean solid whereby the hoop stress σ alters the diameter (subscript θ), length (subscript x) and wall thickness (subscript r) of the glomerular capillaries (Richfield et al., 2021):

$$\sigma_{r}=\frac{E}{3}\varepsilon_{r},$$ (12)

$$\sigma_{\theta }=\frac{E}{3}\varepsilon_{\theta },$$ (13)

$$\sigma_{x}=\frac{E}{3}\varepsilon_{x}.$$ (14)

Where ε denotes the relative change in the glomerular capillary diameter (strain) over the diameter in control conditions, and E is the Young’s modulus of the glomerular capillary walls. Based on data from previous experimental studies wherein glomerular compliance was estimated by quantifying alterations in glomerular volume in response to changes in perfusion pressure (Cortes et al., 1996), we estimate that E for the rat glomerular capillary walls is 14.4 MPa (Richfield et al., 2021). Dividing E by 3 asserts that the wall has the same elasticity in all three directions: axial, radial, and circumferential. In other words, we assume the wall is isotropic, and that the strains are sufficiently small (<10%) to justify a linear stress-strain relationship (Breslavsky et al., 2016). We use this relation to update the diameter, length and thickness of the glomerular capillary walls in response to changes in σ, which allows us to calculate strain of the glomerular capillary walls for a change in arterial pressure. We defer to our previous work (Richfield et al., 2021) for specifics on model implementation.

### Afferent arteriole model

The diameter of the afferent arteriole, denoted D_AA_, changes as a function of the difference between the tension due to blood pressure along the length of the afferent arteriole, denoted T_P_, and the tension produced by the wall as a function of the myogenic and TGF mechanisms, denoted T_wall_ (Carlson et al., 2008; Sgouralis and Layton, 2014). We use the following differential equation to describe these dynamics:

$$\frac{dD_{AA}}{dt}=\frac{1}{\tau_{c}}\left(T_{P}\left(t\right)-T_{wall}\left(t\right)\right),$$ (15)

For τ_c_ constant (Table 1). The tension of the wall due to blood pressure is modeled as

$$T_{P}\left(t\right)=\frac{\left(P_{avg}\left(t\right)-P_{ext}\right)D_{AA}\left(t\right)}{2},$$ (16)

**TABLE 1:** Glomerulus model and systemic parameters. ‘ND’ denotes ‘non-dimensional.’

Parameter	Description	Value	Units	References
k	Capillary hydraulic conductivity	3 × 10^−5^	nl/min/mmHg	Richfield et al. (2020), Richfield et al. (2021)
E	Capillary Young’s modulus	14.4	MPa	
t_GFB_	Capillary wall thickness	360	nm	
μ_pl_	Plasma viscosity	1.24	cP	
C_P_^sys^	Plasma protein concentration	5.94	g/dl	
C_S_^sys^	Plasma osmolality	275	mosmol/kg H_2_O	Navar et al. (1986)
H_t_^sys^	Systemic hematocrit	0.4	ND	
P_A_^0^	Baseline arterial pressure	100	mmHg	
P_BS_^0^	Baseline Bowman’s Space pressure	13	mmHg	

For P_avg_ the average pressure on the length of the afferent arteriole. The interstitial fluid pressure, P_ext_, is assumed to be constant and equal to 5 mmHg (Sgouralis and Layton, 2014).

We calculate P_avg_ given an inlet pressure P_a_ (equal to arterial pressure) by assuming Poiseuille flow in calculating the total afferent resistance R_AA_:

$$R_{AA}\left(t\right)=R_{RA}+\frac{128\mu L_{AA}}{\pi D_{AA}^{4}\left(t\right)},$$ (17)

For R_RA_ a fixed resistance provided by the vessels upstream of the afferent arteriole, μ the apparent viscosity of blood as it traverses the arteriole, and L_AA_ the length of the afferent arteriole (Table 2). In previous autoregulation models (Sgouralis and Layton, 2014) the afferent arteriole blood viscosity is increased roughly ten-fold to provide the resistance necessary to maintain control levels of glomerular blood flow and pressure. In our model we take into account the alteration of blood viscosity as a function of vessel diameter and hematocrit (Pries et al., 1994) (described below), thus we model the afferent arteriole segment within a fixed length L_AA_ upstream from the glomerulus and assume a fixed resistance R_RA_ upstream of this main arteriole segment. The baseline diameter D_AA_^0^ was selected to enforce a specified baseline glomerular blood flow, pressure and SNGFR, as described below.

**TABLE 2:** Autoregulation model parameters gathered from literature. ‘ND’ denotes ‘non-dimensional.’ ‘D’ denotes ‘derived’ parameter, estimated by fitting the model to data.

Parameter	Description	Value	Units	References
D_AA_^0^	Baseline afferent arteriole diameter	7	μm	D
D_EA_^0^	Baseline efferent arteriole diameter	7.3	μm	D
L_AA_	Afferent arteriole length	107	μm	Gattone et al. (1983)
L_EA_	Efferent arteriole length	107	μm	Gattone et al. (1983)
P_ext_	Interstitial pressure	5	mmHg	Sgouralis and Layton (2014)
R_RA_	Pre-afferent arteriolar resistance	1.09	nl mmHg s^−1^	Sgouralis and Layton (2014)
τ_c_	Arteriole diameter rate constant	675	s^−1^	Sgouralis and Layton (2014)
C_act_,_2_	Arteriole constriction shape parameter	0.54	ND	Sgouralis and Layton (2014)

The wall tension, T_wall_ is represented by the sum of a passive tension component, T_pass_ and an active tension component, T_act_ (Carlson et al., 2008):

$$T_{wall}\left(t\right)=T_{pass}\left(t\right)+T_{act}\left(t\right).$$ (18)

The passive tension describes the nonlinear response of the arteriole wall to changes in diameter, independent of the contractile process of the smooth muscle cells (Carlson et al., 2008):

$$T_{pass}\left(t\right)=C_{pass,1}\exp\left(C_{pass,2}\left(\frac{D_{AA}\left(t\right)}{D_{AA}^{0}}-1\right)\right),$$ (19)

Where D_AA_^0^ corresponds to the afferent arteriole diameter at baseline. In general, the superscript 0 indicates the baseline state value, and baseline state values are included in Table 1. The active tension, T_act_ is a sigmoidal function of smooth muscle cell (SMC) tone, denoted S_tone_:

$$T_{act}\left(t\right)=\frac{C_{act,1}}{1+\exp\left(-S_{tone}\left(t\right)\right)}\exp\left(-{\left(\frac{\frac{D_{AA}\left(t\right)}{D_{AA}^{0}}-1}{C_{act,2}}\right)}^{2}\right),$$ (20)

Where C_act,1_ denotes the maximum contractility of the afferent arteriole and C_act,2_ is a shape parameter that describes the nonlinear relationship between afferent contractility and the deviation of D_AA_ from the control state (Sgouralis and Layton, 2014). We model S_tone_ as a linear combination of autoregulatory signals:

$$S_{tone}\left(t\right)=S_{Myo}\left(t\right)+S_{TGF}\left(t\right).$$ (21)

The myogenic mechanism signal (S_Myo_) and TGF signal (S_TGF_) are included. We represent each of these signals as functions of their respective inputs: the myogenic mechanism is modeled as a sigmoid function of the change in afferent arteriole wall tension T_P_ from a reference tension T_P_^ref^ and the TGF mechanism is modeled as a sigmoid function of the change in macula densa concentration C_MD_ from a reference macula densa concentration C_MD_^ref^:

$$S_{Myo}\left(T_{P}\right)=\frac{C_{Myo,\max}}{1+\exp\;\left(-C_{Myo}\left(T_{P}-T_{P}^{ref}\right)\right)}+C_{Myo,\min}$$ (22)

$$S_{TGF}\left(C_{MD}\right)=\frac{C_{TGF,\max}}{1+\exp\;\left(-C_{TGF}\left(C_{MD}-C_{MD}^{ref}\right)\right)}+C_{TGF,\min},$$ (23)

S_tone_ = 0 at baseline, thus S_tone_ is not representative of the absolute magnitude of SMC tone, but the deviation of the SMC tone from baseline, wherein a positive S_tone_ elicits a reduction in D_AA_ from baseline and a negative S_tone_ elicits an enhancement of D_AA_ from baseline.

The baseline afferent and efferent arteriole diameters were derived (reference ‘D’ in Table 2) based on the assumption that at the steady-state control arterial pressure, P_A_^0^ = 100 mmHg, SNGFR, 30 nL/min, P_G_, 50 mmHg, and an afferent arteriole plasma flow of 100 nL/min (blood flow Q_AA_, 166 nL/min) (Navar et al., 1986; Richfield et al., 2020; Richfield et al., 2021). We refer to the interaction between S_Myo_, and S_TGF_, as Ψ, which we discuss in the model parameterization subsection.

### Tubule model

To accurately model TGF responses to changes in perfusion pressure, it is necessary to model tubular fluid flow and osmolality up to the macula densa, taking into account the functional heterogeneity along the nephron’s length. In general, we model the change of osmolality C_T_ on the length of the nephron and the tubular fluid velocity v as:

$$\frac{\partial C_{T}}{\partial t}+v\frac{\partial C_{T}}{\partial x}=-2\pi r_{T}J_{s}$$ (24)

$$\frac{\partial v}{\partial x}=-2\pi r_{T}J_{v}$$ (25)

For v the tubular fluid velocity, r_T_ the tubule radius, J_v_ the volumetric flux, and J_s_ the solute flux defined as:

$$J_{s}=J_{v}\frac{C_{T}}{A}-\frac{P_{s}}{A}\left(C_{T}-C_{e}\right)+\frac{V_{\max}}{A}\frac{C_{T}}{C_{T}+K_{m}},$$ (26)

For A the tubule cross-section. Each term in the right-hand side of Equation (26) represents solute transport by a different mechanism. The first term on the right-hand side of Equation (26) represents the reabsorption of solute due to solute drag, wherein water that is transported across the tubule wall carries dissolved electrolytes through the paracellular channel. The volume flux J_v_ is defined as:

$$J_{v}=P_{v}\left(v\right)\left(C_{T}-C_{e}\right),$$ (27)

Where the volumetric permeability P_v_ is a sigmoid function of the velocity:

$$P_{v}\left(v\right)=\frac{P_{v,c}}{1+\exp\left(-C_{Tv,1}\left(v-C_{Tv,2}\right)\right)}.$$ (28)

The incorporation of a sigmoid curve into the permeability coefficient P_v_ is used to assert glomerulotubular balance such that a lack of flow results in a lack of fluid reabsorption. The constants C_Tv,1_ and C_Tv,2_ control the shape of the sigmoid curve. The volume flux is dependent on the difference between the osmolality in the tubule, C_T_, and the interstitial osmolality, C_e_, where

$$C_{e}\left(x\right)=\left\{\begin{array}{cc} C_{e}^{0}=C_{T}\left(0\right) & 0\leq x<L_{PT}\\ C_{e}^{0}\left(A_{1,DL}\exp\left(\frac{{-A}_{3,DL}\left(x-L_{PT}\right)}{L_{DL}}\right)+A_{2,DL}\right) & L_{PT}\leq x\leq L_{PT}+L_{DL}\\ C_{e}\left(L_{PT}+L_{DL}\right)\left(A_{1,AL}\exp\left(\frac{{-A}_{3,AL}\left(x-L_{PT}-L_{DL}\right)}{L_{AL}}\right)+A_{2,AL}\right) & L_{PT}+L_{DL}<x\leq L_{PT}+L_{DL}+L_{AL} \end{array}\right\}$$ (29)

For L the length of the tubular segment, with subscripts ‘PT,’ ‘DL,’ and ‘AL’ denoting the proximal tubule, descending limb of the Loop of Henle and ascending limb of the Loop of Henle, respectively (Layton et al., 1991). The additional parameters used in these equations are defined as:

$$A_{1,DL}=\frac{1-\frac{C_{e}^{LDL}}{C_{e}^{0}}}{1-\exp\left({-A}_{3,DL}\right)}$$ (30)

$$A_{2,DL}=1-\;A_{1,DL}$$ (31)

$$A_{3,DL}=-2$$ (32)

$$A_{1,AL}=\frac{1-\frac{C_{e}^{LAL}}{C_{e}\left(L_{PT}+L_{DL}\right)}}{1-\exp\left({-A}_{3,AL}\right)}$$ (33)

$$A_{2,AL}=1-A_{1,AL}$$ (34)

$$A_{3,AL}=2.$$ (35)

The notation C_e_ (L_PT_ + L_DL_) refers to a recursive calculation of C_e_ at x = L_PT_ + L_DL_, whereas C_e_^LDL^ and C_e_^LAL^ refer to the constant expected values of interstitial osmolality at the end of the descending and ascending limbs, respectively (Layton et al., 1991).

The second term on the right-hand side of Equation (26) describes passive solute transport (“leak” of solutes back into the tubule) as a function of the difference in osmolality between the tubule lumen and the interstitium (P_s_ is the solute permeability parameter). It is assumed that this passive leak only occurs in the ascending limb, where the volumetric permeability is zero and thus passive leakage of solutes is not ameliorated by solute drag as in the proximal tubule and descending limb. The third term on the right-hand side of Equation (26) corresponds to the active reabsorption of solutes, which is a Michaelis-Menten process with maximum uptake rate denoted by V_max_. The Michaelis-Menten constant, K_m_, is incorporated into this term (Layton et al., 1991).

**FIGURE 2:**
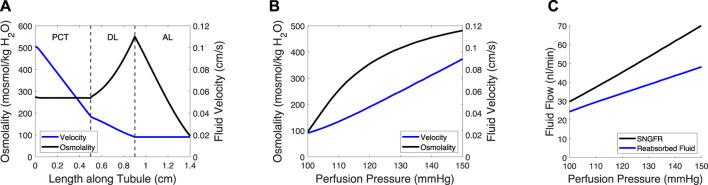
Tubule model predicts osmolality, fluid velocity and the reabsorbed fluid load on the length of the tubule and at varied pressures. **(A)** Tubular fluid osmolality (black) and velocity (blue) as a function of length along the tubule in the baseline case. **(B)** Macula densa osmolality (C_MD_) and fluid velocity at the macula densa as a function of perfusion pressure. **(C)** SNGFR and the reabsorbed fluid load change with altered perfusion pressure. In **(B–C)**, the afferent arteriole and glomerulus models were used to translate perfusion pressure into SNGFR (Figure 1), assuming no feedback (open-loop). The tubule model was then used to compute macula densa osmolality and fluid velocity **(B)**, as well as the reabsorbed fluid load **(C)**.

As stated earlier, each segment of the tubule (assuming three segments: the proximal tubule and the descending and ascending limbs of the Loop of Henle) exhibit different transport processes. This is modeled by altering the parameters depending on the tubular segment (Table 3). Equations 24 and 25 are solved for each segment of the tubule, with the concentration and velocity at the end of the segment acting as the boundary conditions for the next segment (Figure 2). In the steady-state condition, Equations 24 and 25 become:

$$\frac{dC_{T}}{dx}=-2\pi \frac{r_{T}J_{s}}{v}$$ (36)

$$\frac{dv}{dx}=-2\pi r_{T}J_{v}.$$ (37)

**TABLE 3:** Tubule model parameters. ‘D’ denotes ‘derived’ parameter from data.

Parameter	Description	Tubule segment	Value	Units	References
r_T_	Tubule radius	PCT	12.5	μm	Weinstein (1986)
		DL	12.5		Layton et al. (2012)
		AL	10		Layton et al. (1991)
L_T_	Tubule length	PCT (L_PT_)	0.5	cm	Layton et al. (2012)
		DL (L_DL_)	0.4		Sgouralis and Layton (2014)
		AL (L_AL_)	0.5		Layton et al. (2012)
P_S_	Passive solute permeability	PCT	0	μm s^−1^	D
		DL	0		D
		AL	0.15		Layton et al. (2012)
V_max_	Maximum active transport rate	PCT	28	nmol cm^−2^ s^−1^	Layton et al. (2012)
		DL	0		Layton et al. (2012)
		AL	16.6		Layton et al. (2012)
C_Tv,1_	Glomerulotubular balance parameters		350	s cm^−1^	D
C_Tv,2_			0.03	cm s^−1^	D
P_v,c_	Volumetric permeability	PCT	0.15	μm s^−1^ (mOsmol/kg H_2_O)^−1^	D
		DL	0.15		D
		AL	0		Layton et al. (1991)
K_m_	Michaelis-Menten constant		70	mOsmol/kg H_2_O	Layton et al. (1991)
C_e_^LDL^	Interstitial osmolality at loop bend		650	mOsmol/kg H_2_O	Knepper et al. (2003)
C_e_^LAL^	Interstitial osmolality at the macula densa		150	mOsmol/kg H_2_O	Layton et al. (1991)

The glomerulotubular balance parameters C_Tv,1_ and C_Tv,2_ were estimated to ensure that Equation (35) remained stable at low SNGFR values, wherein the tubular fluid velocity approached 0. We assumed that there was 0 effective passive solute permeability in the proximal tubule (PCT) and distal limb of the Loop of Henle (DL), because the meager impact of passive transcellular transport is dwarfed by the active transport of solutes and solute drag (García et al., 1998). We then adjusted the volumetric permeability P_v,c_ of the PCT and DL until the fluid flow at the loop bend equaled ∼20% of SNGFR (Layton et al., 1991; Layton et al., 2012) and the C_MD_ = 100 mosmol/kg H_2_O (Bell and Navar, 1982; Navar et al., 1982), at baseline.

### Renal autoregulation model algorithm

The afferent arteriole, glomerulus and tubule models were linked in series, wherein each model fed into the next model, creating a feedback loop (Figure 1). In the model, an input pressure (equal to arterial pressure, assumed 100 mmHg at baseline), is translated into an input pressure and flow for the glomerulus model. The glomerulus model calculates the filtered volume of fluid (SNGFR), which is input for the tubule model. The tubule model calculates the osmolality at the macula densa, denoted C_MD_. This osmolality and the tension of the afferent arteriole are converted into autoregulatory signals S_TGF_ and S_Myo_, respectively (equations 22 and 23). These are summed and used to modify the afferent arteriole diameter. This feedback loop can be used to simulate transient changes in afferent arteriole diameter, however, we trained our model on steady-state data, as described below. As such, the model was solved assuming that the afferent arteriole diameter is steady-state, and

$$T_{P}=T_{wall}.$$ (38)

This assumption implies that the insights we gained from our model are limited to the steady-state stresses exerted on the glomerular capillaries, and the mechanical stresses/strains that these capillaries undergo in transient changes in blood pressure that occur at high speeds. We assumed that we could estimate the latter under steady-state conditions because at the frequency of a rat heartbeat (400 Hz), afferent arterioles are unlikely to change their diameter; studies by Walker et al. showed that, in response to a rapid step in pressure, afferent arterioles take over a minute to fully respond (Walker et al., 2000; Walker, 2001). Any spontaneous oscillations of the afferent arteriole occur at a frequency more than two magnitudes lower than the rat heart rate (Holstein-Rathlou, 1987; Holstein-Rathlou and Marsh, 1990; Holstein-Rathlou and Marsh, 1994; Marsh et al., 2005a; Marsh et al., 2005b). Thus, after parameterizing our model, we used it to evaluate the impact of the autoregulatory mechanisms on transient and steady-state mechanical stresses exerted on the glomerular capillary walls.

### Renal autoregulation model parameterization

The mathematical model was partially parameterized using afferent arteriole blood flow data gathered from a previous study (Takenaka et al., 1994). In this study, the juxtamedullary nephron preparation was used to investigate the impact of myogenic and TGF mechanisms on afferent arteriole diameter. A high dose of furosemide was administered to block TGF activity; the same experiment was performed with a papillectomy as the intervention (essentially guaranteeing a loss of TGF), which showed the same results. Additionally, diltiazem, a calcium channel blocker, was used to negate both the TGF and myogenic mechanisms. Using the juxtamedullary nephron preparation, and under each of these experimental conditions, steady-state perfusion pressure was increased from 100 mmHg (baseline) to 150 mmHg, and steady-state afferent arteriole diameter and blood flow were measured.

**FIGURE 3:**
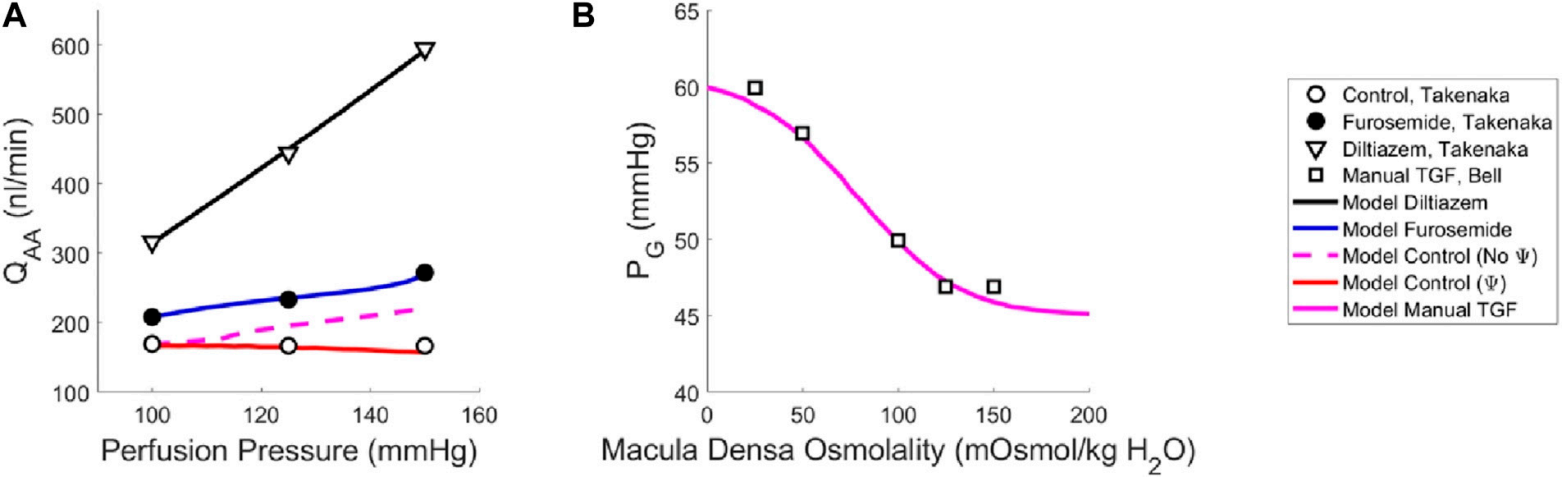
Renal autoregulation model parameterization. Data obtained from literature from Takenaka (Takenaka et al., 1994) and Bell (Bell and Navar, 1982) are represented as points, wherein **(A)** open circles indicate the control animals, closed circles indicate the animals that received furosemide, open triangles indicate animals that received diltiazem, and **(B)** open squares indicate animals whose TGF response was manually controlled by placing a wax block in the proximal tubule. Model results are shown as curves, **(A)** black indicating a passive afferent arteriole, blue indicating only the myogenic mechanism is active, dashed magenta indicates both myogenic and TGF mechanisms are active but do not interact (no Ψ), red indicates that both mechanisms are operant and that the myogenic mechanism sensitivity is modified by the TGF (Ψ). **(B)** The solid magenta line indicates that the myogenic mechanism is active, but TGF is manually controlled (i.e., tubular fluid concentration is stable, without TGF operant).

Our model of renal autoregulation was fit to this data (Figure 3) by using each experimental group as a limit case: the diltiazem data (3 datapoints) were used to estimate the passive parameters C_pass,1_ and C_pass,2_ (2 parameters) by setting C_Act_ = 0. The maximum active contractility C_Act,1_ was estimated assuming S_tone_ = 0 in the baseline case and solving Equation (15) assuming steady-state conditions (1 data point, 1 parameter). The Takenaka, 1994 furosemide data (3 data points) were used to estimate the myogenic mechanism parameters C_Myo_, T_P_^ref^, C_Myo,max_ and C_Myo,min_ (4 parameters), by setting S_TGF_ = 0. To estimate 4 parameters from 3 data points, we assumed that the minimum and maximum S_Myo_ values occurred at pressures of 80 and 180 mmHg, respectively. By assuming that the sigmoid S_Myo_ curve is approximately linear between 80 and 180 mmHg, we estimated C_Myo,max_ and C_Myo,min_ as well as T_P_^ref^ using this assumption, and finally fit C_Myo_ to the original 3 datapoints. Parameterization was performed by minimizing the least squared error between the model Q_AA_ and the corresponding Takenaka blood flow data under each of the experimental conditions. To ameliorate issues involving discrepancies between animal and mathematical model baseline state pressure and flow values, the model was fit to the Q_AA_ values relative to the model baseline. Importantly, the diltiazem case assumed that the efferent arteriole diameter, as a function of diltiazem concentration, increases at a rate equal to 20% of that of the afferent arteriole as a function of diltiazem dose (Hayashi et al., 2003).

To estimate the TGF mechanism parameters, we used data from a different study that measured stop flow pressure changes in response to alterations in tubular osmolality (Bell and Navar, 1982). In this experiment, the myogenic mechanism was operant but the macula densa was cut off from the glomerulus due to a wax block placed in the proximal tubule. As a result, the feedback-isolated TGF response to manual changes in tubular osmolality could be measured in the form of changes in stop flow pressure. Model parameters C_TGF_, C_MD_^ref^, C_TGF,max_ and C_TGF,min_ (4 parameters) were fit to this data (5 data points) by estimating the change in afferent arteriole diameter that mediates the changes in glomerular pressure seen experimentally (Figure 3B).

Once we parameterized the steady-state TGF and myogenic signals, S_TGF_ and S_Myo_, respectively, we compared the model output to that of the control condition animals from Takenaka (Takenaka et al., 1994), and showed that the addition of S_TGF_ and S_Myo_ (as in Eq. (21)) correctly estimates the control state afferent arteriole diameter at baseline perfusion pressure (Figure 3, dashed magenta). This serves as verification that our model was properly parameterized. However, at higher pressures, the model fails to reproduce the experimental results, as it is unable to constrict to the point where flow is maintained at homeostatic levels. In the juxtamedullary nephron preparation, there is no endocrine signaling to the vasculature and we are not aware of local, paracrine feedback mechanisms that could mediate this constriction other than TGF and the myogenic response. As such, we assumed that the tone required to generate the constrictive response at higher perfusion pressures is generated by the TGF mechanism’s modulation of the myogenic mechanism sensitivity (Walker et al., 2000; Walker, 2001; Cupples, 2007; Scully et al., 2013; Scully et al., 2016; Scully et al., 2017) which we denote Ψ. Namely, we define Ψ as the new set of parameters C_Myo_ and T_P_^ref^ that only are used if TGF is operant:

$$\Psi =\left.\left\{C_{Myo},T_{P}^{ref}\right.\right|\;S_{TGF}\left.\neq 0\right\}$$ (39)

**FIGURE 4:**
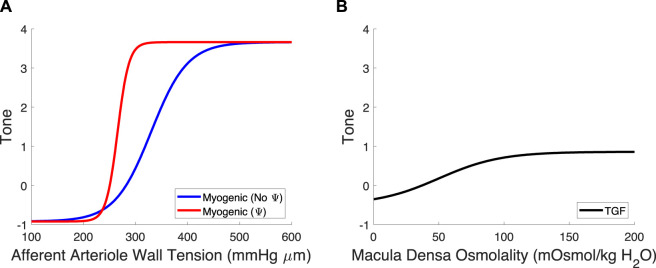
Myogenic mechanism and TGF signal curves. **(A)** The myogenic curve (blue) is a function of the afferent arteriole wall tension. The TGF-mediated modification to the myogenic curve, in red, is included to show the difference between the myogenic mechanism with and without TGF operant. **(B)** The TGF curve is black and is a function of the macula densa osmolality.

We recalculated values for C_Myo_ and T_P_^ref^ to create a new myogenic curve (red in Figure 4, entries in Table 4) that fits the control data generated by Takenaka et al. (Takenaka et al., 1994) and calculates S_tone_ as in Equation (21). We fit C_Myo_ and T_P_^ref^ (2 parameters) to the Takenaka control data (3 data points). We included these values in Table 4, distinguishing them from the parameter values associated with the myogenic curve when TGF is inoperant (see Ψ column). By altering these parameters (increasing the slope and shifting the myogenic curve left in Figure 4), the model fits the control data from (Takenaka et al., 1994) and shows adequate control of blood flow Q_AA_ (Figure 3). We quantify the model error in supplementary table S1. Because each of the eleven unknown parameters were separately estimated by datasets that contained the same number of datapoints if not more datapoints than parameters, we assume that the model is identifiable.

**TABLE 4:** Autoregulation model parameters fit to data from literature (Bell and Navar, 1982; Takenaka et al., 1994).

Parameter	Ψ	Value	Units	Source data	NSC, 100 mmHg	NSC, 130 mmHg
C_Pass,1_	-	83.0	μm mmHg	(Takenaka et al., 1994), diltiazem	−0.06	−0.03
C_Pass,2_	-	4.41	ND	(Takenaka et al., 1994), diltiazem	0	0.02
C_Act,1_	-	310	μm mmHg	(Takenaka et al., 1994), control	−0.12	−0.15
T_P_^ref^	No	330	μm mmHg	(Takenaka et al., 1994), furosemide	0.60	0.97
	Yes	265	μm mmHg	(Takenaka et al., 1994), control		
C_Myo_	No	0.029	μm−1 mmHg−1	(Takenaka et al., 1994), furosemide	0.05	0.03
	Yes	0.092	μm−1 mmHg−1	(Takenaka et al., 1994), control		
C_Myo,max_	-	4.58	ND	(Takenaka et al., 1994), furosemide	−0.03	−0.06
C_Myo,min_	-	−0.92	ND	(Takenaka et al., 1994), furosemide	0.05	0.04
C_TGF,max_	-	1.06	ND	Bell and Navar (1982)	−0.06	−0.03
C_TGF,min_	-	−0.200	ND	Bell and Navar (1982)	0.03	0.02
C_TGF_	-	0.042	(mosmol/kg H2O)−1	Bell and Navar (1982)	−0.06	0
C_MD_^ref^	-	50.0	mosmol/kg H2O	Bell and Navar (1982)	0.02	0.02

### Model code

All data analysis, model parameterization and model code were written in R version 3.5.1, with simulations performed on a personal laptop computer. Results were visualized and figures generated using MatLab 2022. All code and data is available in a public GitHub repository: https://github.com/omrichfield/autoreg_glommod. We have released the repository with DOI 10.5281/zenodo.11114851.

## Results

### Steady-state glomerular hemodynamics

**FIGURE 5:**
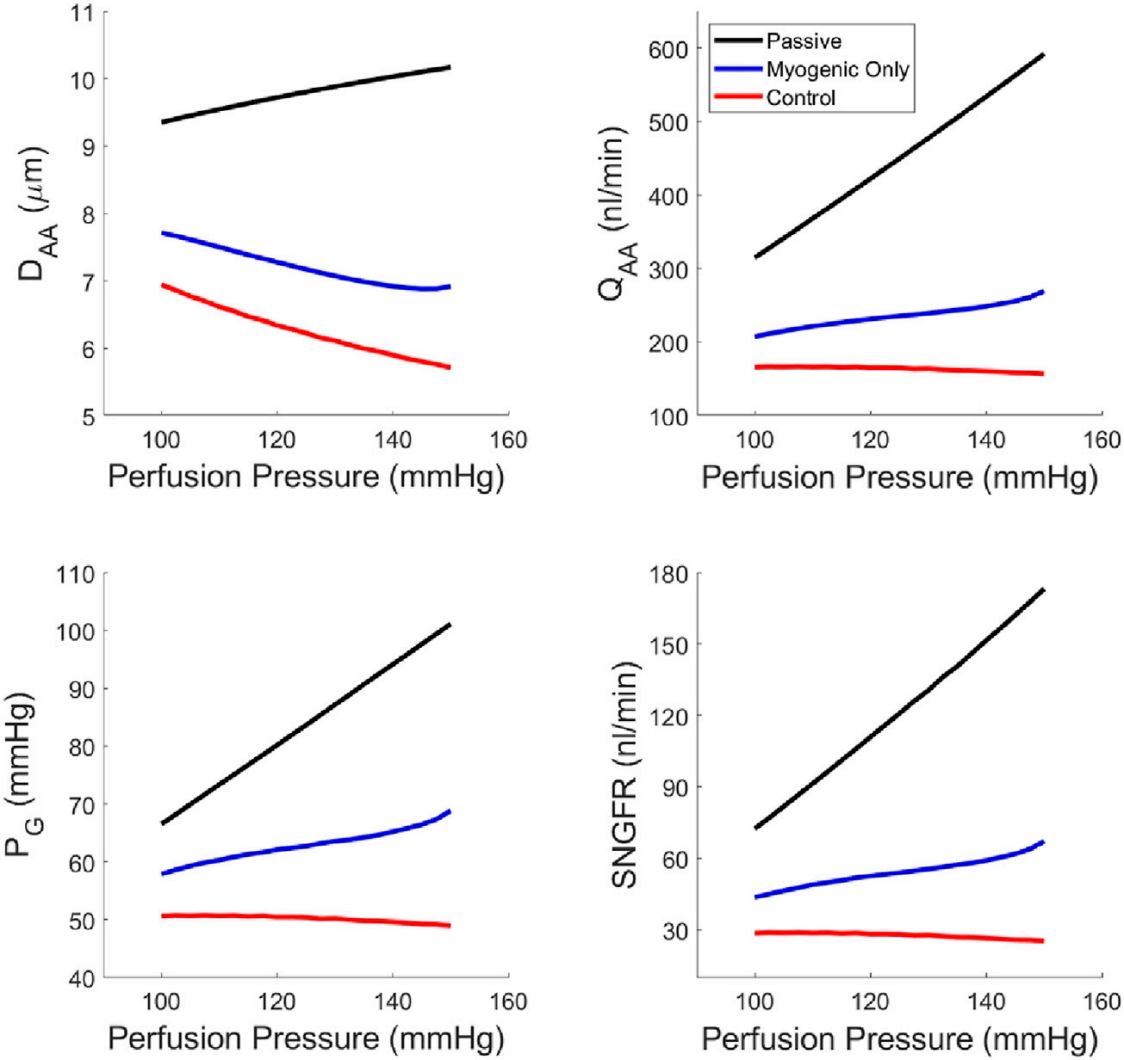
Steady-state glomerular hemodynamics with each autoregulatory mechanism removed to show the functional results of reduction in autoregulatory efficiency.

Using our newly parameterized autoregulation model, we estimated functional readouts (afferent arteriole diameter D_AA_, blood flow Q_AA_, SNGFR and glomerular pressure P_G_) under passive conditions, with only the myogenic mechanism operant, and the control condition with TGF, the myogenic mechanism and their interaction, Ψ (Figure 5). The addition of TGF serves to reduce the baseline diameter but does not enhance the reduction in diameter in response to an increasing perfusion pressure. As a result, only when both mechanisms are operant and Ψ is present, are P_G_ and SNGFR maintained at baseline levels despite increasing perfusion pressure.

### Sensitivity analysis

We computed the normalized sensitivity coefficient (NSC) for each of the autoregulation model parameters at both P_A_ = 100 mmHg and 130 mmHg (Table 4). NSC for a parameter P is calculated as:

$$NSC=\frac{\frac{Q_{AA}^{1}-Q_{AA}^{0}}{Q_{AA}^{0}}}{\frac{P^{1}-P^{0}}{P^{0}}}$$ (40)

Where P^0^ indicates the normal parameter value and P^1^ is equal to 101% of P^0^. Q_AA_^x^ indicates the afferent arteriole blood flow for when P=P^x^ for x = 0, 1. Thus, NSC measures the relative change in afferent arteriole blood flow given a 1% increase in the parameter P. An |NSC| > 0.5 is considered significant, and |NSC| < 0.5 is considered insignificant. Accordingly, the only parameter that shows significant model sensitivity is T_P_^ref^ (Table 4).

### Glomerular mechanics

**FIGURE 6:**
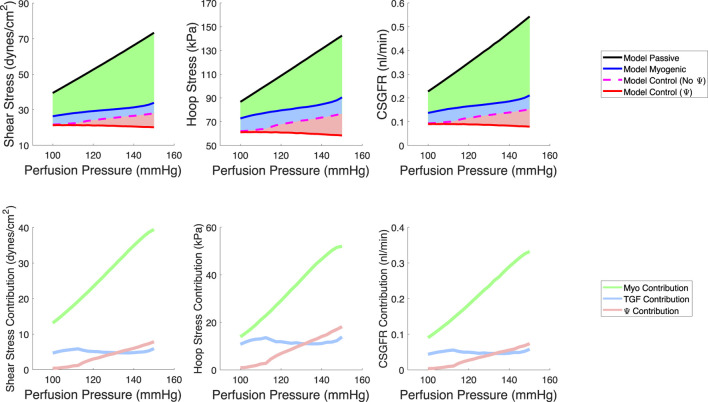
Model predictions of steady-state, spatially averaged shear stress, hoop stress and CSGFR values generated by a varied perfusion pressure (top row). We model a passive afferent arteriole (black), an afferent arteriole with only the myogenic mechanism operant (blue), the additive TGF and myogenic mechanisms (dashed magenta, no Ψ), and the TGF with a modified myogenic mechanism (red, with Ψ). We then compute the contribution of each autoregulatory mechanism to the maintenance of the mechanical stresses at control values (bottom row). The myogenic mechanism contribution (green) is highest, while TGF (blue) and Ψ (pink) play a smaller role in contributing to the maintenance of glomerular mechanical homeostasis.

**FIGURE 7:**
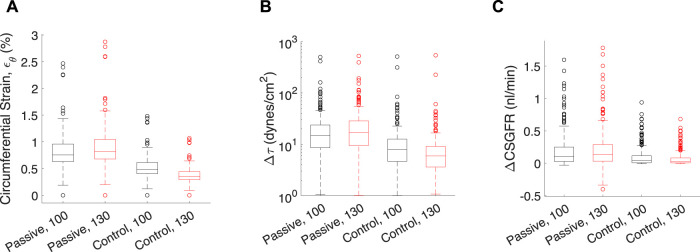
Transient mechanical stress and strains exerted on each glomerular capillary by a pressure pulse from the rat heartbeat. Each data point corresponds to one of the 320 glomerular capillaries in the network. Transient mechanical stresses include capillary circumferential strain (ε_θ_, **(A)**, change in shear stress on the endothelium (Δτ, **(B)** and the change in CSGFR (ΔCSGFR, **(C)**. The model afferent arteriole parameters are varied to simulate the passive (‘Passive’), and the control condition with Ψ (‘Control’). Two mean perfusion pressures (P_A_) were considered, 100 mmHg (black boxes) and 130 mmHg (red boxes), as indicated by the number next to the model condition for each group. Lines in each box indicate the median across all capillaries, the bottom and top of the boxes indicate 75th and 25th percentiles, respectively, and whiskers indicate outliers.

We quantified the magnitudes of mechanical stress in the glomerulus under steady-state conditions (Figure 6) as well as during transient changes in blood pressure characteristic of physiological conditions (Figure 7). By testing different autoregulatory scenarios (passive, myogenic only, TGF and myogenic and TGF with the modified myogenic), we estimated the contribution Ω of each autoregulatory mechanism to the maintenance of glomerular mechanical homeostasis. Three mechanical stresses were considered; shear stress against the endothelial cells, hoop stress on the podocytes, and the CSGFR, which we assume is proportional to the shear stress experienced by the podocytes during filtration. We define the steady-state mechanical stress contributions as follows, where M denotes the mechanical stress (shear, hoop, CSGFR) and the subscript ‘Pass,’ ‘Myo,’ ‘TGF,’ and ‘Ψ’ denote the cases of passive afferent arteriole, myogenic mechanism only, additive myogenic and TGF, and TGF with the TGF-modulated myogenic mechanism, respectively.

$$\left.\Omega_{Myo}\left.=\right|M_{Pass}\left(P_{A}\right)-M_{Myo}\left(P_{A}\right)\right|$$ (41)

$$\left.\Omega_{TGF}\left.=\right|M_{Myo}\left(P_{A}\right)-M_{TGF}\left(P_{A}\right)\right|$$ (42)

$$\left.\Omega_{\Psi }\left.=\right|M_{TGF}\left(P_{A}\right)-M_{\Psi }\left(P_{A}\right)\right|$$ (43)

The respective contributions of each mechanism to the maintenance of steady-state mechanical stress in the glomerulus are shown on the bottom row of Figure 6.

In addition to steady-state values of shear stress, hoop stress and CSGFR, we computed the transient changes in shear stress and CSGFR (denoted Δτ and ΔCSGFR, respectively), along with the circumferential strain (ε_θ_), generated by the rat heartbeat. We considered two mean blood pressures, 100 mmHg and 130 mmHg, with an oscillation amplitude of 20 mmHg. Since the rat’s heartbeat occurs at a frequency of 400 Hz, we assume that during the average transient change in blood pressure associated with diastole and systole of the rat’s heart, the afferent arteriole diameter is constant (Walker et al., 2000; Walker, 2001). We computed the change in shear stress, CSGFR and glomerular capillary diameter (strain, ε_θ_) associated with a fluctuation in blood pressure from 80 mmHg to 120 mmHg (with a mean of 100 mmHg) and 110 mmHg–150 mmHg (with a mean of 130 mmHg), for each glomerular capillary in the network (Richfield et al., 2021) (Figure 7). We considered both the passive case and the case of complete autoregulation, and see that complete amelioration of autoregulation increases the baseline (100 mmHg mean blood pressure) mechanical stresses, and also fails to control mechanical stresses when blood pressure is elevated (130 mmHg mean blood pressure).

**FIGURE 8:**
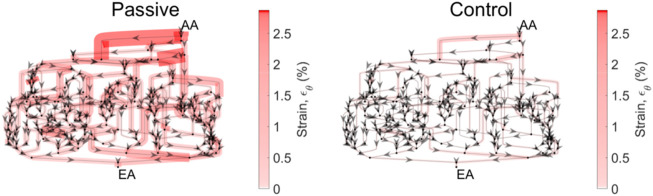
Circumferential strain distribution across the glomerular capillary network. Each of the networks above is representative of the network topology of the model glomerulus; each segment represents a capillary segment, with nodes connecting the segments as a representation of points of bifurcation and/or coalescing. Arrows indicate flow direction. The thickness of the capillary, as well as the color (scale bar at right) are proportional to the circumferential strain on that capillary segment. Mean pressure is 130 mmHg. The strain is calculated for the passive case, when both autoregulatory mechanisms are inoperant, and the control case, in which both mechanisms are operant and their interaction Ψ is present.

Apparent in Figure 7 is the variability of the mechanical stresses and strains throughout the glomerular capillary network; for example, at baseline conditions, some capillaries exhibit 1.5% wall strain while the median across all capillaries in the network is 0.5%. We have used our glomerulus model to estimate this heterogeneity previously (Richfield et al., 2020; Richfield et al., 2021). To better characterize the spatial location of the vessels at greatest risk of mechanical damage in the event of loss of renal autoregulation, we mapped the strain of the glomerular capillaries on a graphical model of the rat glomerulus (Figure 8).

From the spatial analysis in Figure 8, it is clear that the attenuation of renal autoregulation—wherein the afferent arteriole is passive, as opposed to the afferent arteriole with full autoregulatory capability, doubles the strain of some of the vessels nearest to the afferent arteriole. This is the case for perfusion pressure at 130 mmHg, wherein the median strain increases by 50% with the total loss of autoregulation (Figure 7).

## Discussion

We developed a novel model of renal autoregulation that estimates how each mechanism of autoregulation contributes to changes in glomerular filtration and mechanics at the capillary level. Building on previous renal autoregulation modeling studies, our model is unique in that it quantitatively estimates the mechanical stresses and local filtration dynamics in an anatomically accurate glomerular capillary network. Our results indicate that the reduction of renal autoregulatory efficiency by the attenuation of the autoregulatory mechanisms and their interaction significantly increases the magnitude of mechanical stresses exerted on the glomerular cells. These results become more apparent as blood pressure is elevated, highlighting the importance of renal autoregulatory mechanisms in the maintenance of glomerular mechanical homeostasis in hypertensive conditions.

One key finding of this study is that to properly predict the baseline autoregulatory response to elevated blood pressure, our model indicates that the myogenic mechanism sensitivity is heightened when TGF is operant. We reached this conclusion by parameterizing the model using two separate experimental datasets and found that the myogenic mechanism sensitivity must be heightened when TGF is operant, to adequately maintain glomerular function as perfusion pressure is increased. Numerous studies have posited that TGF signaling modulates the sensitivity of the myogenic mechanism (Walker et al., 2000; Walker, 2001; Cupples, 2007; Scully et al., 2013; Mitrou et al., 2015; Scully et al., 2016; Scully et al., 2017). In particular, Walker et al. showed that the steady-state and dynamic myogenic responses to increased perfusion pressure are more robust when TGF is operant as opposed to when TGF is attenuated using a large dose of furosemide (Walker et al., 2000). As in these studies, we quantify the modulatory relationship between TGF and myogenic mechanisms, denoted Ψ, by measuring the difference between the myogenic curve parameters T_P_^ref^ and C_Myo_ with and without TGF operant. Our analysis suggests that, by shifting the myogenic curve leftward on the wall tension-axis, TGF heightens the sensitivity of the myogenic mechanism.

Our model of renal autoregulation is new as compared to previous autoregulation models in that it estimates the mechanical stresses and individual filtration rates for each capillary in the glomerular capillary network. Pairing this model with an afferent arteriole model enabled us to model the glomerular capillary mechanics under different autoregulatory conditions. We used this novel model to quantify the contribution of each autoregulatory mechanism to the maintenance of glomerular mechanical homeostasis. We calculated the steady-state hoop stress, shear stress and CSGFR averaged across the capillaries in the network. As anticipated, with the addition of each autoregulatory mechanism (myogenic, TGF, and their interaction Ψ), mechanical stresses were reduced. This became more apparent as perfusion pressure was increased. In addition to evaluating the impact of each autoregulatory mechanism on glomerular mechanics as a function of perfusion pressure, we also investigated the impact of the autoregulatory mechanisms on the mechanical stress magnitudes in different glomerular capillaries within the network (Figures 7, 8). As expected, a passive afferent arteriole transmits a larger strain and shear stress to the glomerular capillaries when blood pressure is elevated, while activation of both mechanisms and their interaction controls the mechanical stresses at baseline levels.

What is apparent from Figure 8 is the heterogeneity of mechanical stresses and strains exerted throughout the glomerular capillary network, as we have described previously (Richfield et al., 2020; Richfield et al., 2021). Our previous work has identified strain as a potential factor in the progression of glomerular injury in hypertension, diabetes and severe chronic kidney disease, all of which involve glomerular hypertension. This suggests that these capillaries are most susceptible to mechanical dysregulation as autoregulatory control is attenuated. As we have noted previously, damage to the capillaries nearest to the afferent arteriole fits the pattern of perihilar glomerulosclerosis seen clinically, secondary to significant loss of functional nephron mass and high blood pressure (Fogo, 2015). Thus, our results generate the hypothesis that mechanical injury to the glomerular cells may play a role in the progression of glomerulosclerosis under these conditions.

Podocytes reorganize their actin cytoskeleton to maintain attachment when exposed to shear stress *in vitro* (Friedrich et al., 2006), and, when subjected to 5%–7% biaxial strain, podocytes hypertrophy (Petermann et al., 2005) and reorganize their actin cytoskeleton to accommodate the enhanced mechanical load (Endlich et al., 2001; Endlich et al., 2002). As the maximum strain magnitude computed by our model is in the perihilar glomerular capillaries and is equal to 3%, our model suggests that the loss of autoregulation is not sufficient to cause strain that podocytes will need to remodel and thereby perpetuate sclerosis. Other insults such as enhanced blood pressure and/or a reduction in podocyte stiffness may be required to cause the damage seen experimentally. Other mechanical stresses, such as shear stress on the podocyte, are not exerted on podocytes in *vitro* stretch studies, thus the combination of different mechanical stresses may play a role in podocyte injury when autoregulation is attenuated.

As with all mathematical models, simulation results must be contextualized within the assumptions and limitations of the mathematical model design. The limitations of the model defined here are principally derived from the limitations in the data used to parameterize the model (Bell and Navar, 1982; Takenaka et al., 1994); both datasets used to parameterize the model were collected at steady-state, thus model insights are limited to the case when the afferent arteriole diameter is constant (i.e., Equation (38) is satisfied). In this study, we estimated the steady-state mechanical stresses (Figure 6) and transient mechanical stresses that are assumed to occur so quickly that the afferent arteriole diameter is assumed constant during this period (Figures 7, 8). We did not consider the case in which mechanical stresses may change over the course of an interim time period, on the order of seconds or minutes. The limitation of the current study suggests further research directions.

In conclusion, we developed a novel model of renal autoregulation that incorporates an anatomically accurate mathematical model of blood flow and filtration in a rat glomerulus, to estimate how the autoregulatory mechanisms (TGF and myogenic) control glomerular mechanical stress magnitudes. Our model results indicate that at high perfusion pressure, an interaction between TGF and myogenic mechanisms is required to maintain glomerular pressure, filtration, and mechanical stresses at baseline levels. Using our mathematical model of the glomerular capillary network, we quantified mechanical stress magnitudes throughout the population of capillaries and identified the ‘perihilar’ capillaries that branch off the afferent arteriole as potential sites of mechanical injury to podocytes. This study highlights the utility of mathematical models in examining complex physiological questions that are difficult or impossible to measure experimentally.

## Data Availability

The data and mathematical models used to support the findings of this study are available on GitHub at https://github.com/omrichfield/autoreg_glommod and on Zenodo at https://doi.org/10.5281/zenodo.11114851.

## Appendix A

**TABLE A1:** Model error quantification. Model afferent arteriole blood flows (blue) are compared to afferent arteriole blood flow Q_AA_ measured experimentally under different pharmacological conditions (green), at three perfusion pressures. Error is calculated as a percentage of the experimental value.

Pressure (mmHg)	100	125	150
Model Diltiazem	315	450	592
Diltiazem	317	444	594
% error	0.63	1.30	0.34
Model Furosemide	208	236	270
Furosemide	208	233	272
% error	0.0	1.3	0.74
Model Control (No Ψ)	167	196	221
Control	169	167	167
% error	1.2	17.4	32.3
Model Control (Ψ)	166	165	157
Control	169	167	167
% error	1.8	1.2	6.0

## References

[B1] Bell P. D. Navar L. G. (1982). Relationship between tubulo-glomerular feedback responses and perfusate hypotonicity. Kidney Int. 22 (3), 234–239. 10.1038/ki.1982.160 7176326

[B2] Breslavsky I. D. Amabili M. Legrand M. (2016). Static and dynamic behavior of circular cylindrical shell made of hyperelastic arterial material. J. Appl. Mech. 83 (5). 10.1115/1.4032549

[B3] Carlson B. E. Arciero J. C. Secomb T. W. (2008). Theoretical model of blood flow autoregulation: roles of myogenic, shear-dependent, and metabolic responses. Am. J. Physiology-Heart Circulatory Physiology 295 (4), H1572–H1579. 10.1152/ajpheart.00262.2008 PMC2593503 18723769

[B4] Carlson B. E. Secomb T. W. (2005). A theoretical model for the myogenic response based on the length–tension characteristics of vascular smooth muscle. Microcirculation 12 (4), 327–338. 10.1080/10739680590934745 16020079

[B5] Ciocanel M.-V. Stepien T. Sgouralis I. Layton A. (2018). A multicellular vascular model of the renal myogenic response. Processes 6 (7), 89. 10.3390/pr6070089

[B6] Cortes P. Zhao X. Riser B. L. Narins R. G. (1996). Regulation of glomerular volume in normal and partially nephrectomized rats. Am. J. Physiology-Renal Physiology 270 (2), F356–F370. 10.1152/ajprenal.1996.270.2.F356 8779898

[B7] Cupples W. A. (2007). Interactions contributing to kidney blood flow autoregulation. Curr. Opin. Nephrol. Hypertens. 16 (1), 39–45. 10.1097/MNH.0b013e3280117fc7 17143070

[B8] Deen W. Robertson C. Brenner B. (1972). A model of glomerular ultrafiltration in the rat. Am. J. Physiology-Legacy Content 223 (5), 1178–1183. 10.1152/ajplegacy.1972.223.5.1178 4654350

[B9] Edwards A. Layton A. T. (2014). Calcium dynamics underlying the myogenic response of the renal afferent arteriole. Am. J. Physiology-Renal Physiology 306 (1), F34–F48. 10.1152/ajprenal.00317.2013 PMC3921822 24173354

[B10] Endlich K. Kliewe F. Endlich N. (2017). Stressed podocytes—mechanical forces, sensors, signaling and response. Pflügers Archiv-European J. Physiology 469 (7-8), 937–949. 10.1007/s00424-017-2025-8 28687864

[B11] Endlich N. Endlich K. (2012). “The challenge and response of podocytes to glomerular hypertension,” in Seminars in nephrology (Elsevier). 10.1016/j.semnephrol.2012.06.004 22958487

[B12] Endlich N. Kress K. R. Reiser J. Uttenweiler D. Kriz W. Mundel P. (2001). Podocytes respond to mechanical stress *in vitro* . J. Am. Soc. Nephrol. 12 (3), 413–422. 10.1681/ASN.V123413 11181788

[B13] Endlich N. Sunohara M. Nietfeld W. Wolski E. W. Schiwek D. Kränzlin B. (2002). Analysis of differential gene expression in stretched podocytes: osteopontin enhances adaptation of podocytes to mechanical stress. FASEB J. 16 (13), 1850–1852. 10.1096/fj.02-0125fje 12354696

[B14] Fogo A. B. (2015). Causes and pathogenesis of focal segmental glomerulosclerosis. Nat. Rev. Nephrol. 11 (2), 76–87. 10.1038/nrneph.2014.216 25447132 PMC4772430

[B15] Franco M. Bautista R. Tapia E. Soto V. Santamaría J. Osorio H. (2011). Contribution of renal purinergic receptors to renal vasoconstriction in angiotensin II-induced hypertensive rats. Am. J. Physiology-Renal Physiology 300 (6), F1301–F1309. 10.1152/ajprenal.00367.2010 PMC3119140 21367914

[B16] Friedrich C. Endlich N. Kriz W. Endlich K. (2006). Podocytes are sensitive to fluid shear stress *in vitro* . Am. J. Physiology-Renal Physiology 291 (4), F856–F865. 10.1152/ajprenal.00196.2005 16684926

[B17] García N. H. Ramsey C. R. Knox F. G. (1998). Understanding the role of paracellular transport in the proximal tubule. Physiology 13 (1), 38–43. 10.1152/physiologyonline.1998.13.1.38 11390757

[B18] Gattone V. H. 2nd Evan A. P. Willis L. R. Luft F. C. (1983). Renal afferent arteriole in the spontaneously hypertensive rat. Hypertension 5 (1), 8–16. 10.1161/01.hyp.5.1.8 6848472

[B19] Hayashi K. Ozawa Y. Fujiwara K. Wakino S. Kumagai H. Saruta T. (2003). Role of actions of calcium antagonists on efferent arterioles – with special references to glomerular hypertension. Am. J. Nephrol. 23 (4), 229–244. 10.1159/000072054 12840599

[B20] Holstein-Rathlou N. Marsh D. (1990). A dynamic model of the tubuloglomerular feedback mechanism. Am. J. Physiology-Renal Physiology 258 (5), F1448–F1459. 10.1152/ajprenal.1990.258.5.F1448 2337158

[B21] Holstein-Rathlou N.-H. (1987). Synchronization of proximal intratubular pressure oscillations: evidence for interaction between nephrons. Pflügers Arch. 408 (5), 438–443. 10.1007/BF00585066 3601634

[B22] Holstein-Rathlou N.-H. Marsh D. J. (1994). Renal blood flow regulation and arterial pressure fluctuations: a case study in nonlinear dynamics. Physiol. Rev. 74 (3), 637–681. 10.1152/physrev.1994.74.3.637 8036249

[B23] Kasiske B. L. O’Donnell M. P. Garvis W. J. Keane W. F. (1988). Pharmacologic treatment of hyperlipidemia reduces glomerular injury in rat 5/6 nephrectomy model of chronic renal failure. Circulation Res. 62 (2), 367–374. 10.1161/01.res.62.2.367 3338121

[B24] Knepper M. A. Saidel G. M. Hascall V. C. Dwyer T. (2003). Concentration of solutes in the renal inner medulla: interstitial hyaluronan as a mechano-osmotic transducer. Am. J. Physiology-Renal Physiology 284 (3), F433–F446. 10.1152/ajprenal.00067.2002 12556362

[B25] Kriz W. Lemley K. V. (2015). A potential role for mechanical forces in the detachment of podocytes and the progression of CKD. J. Am. Soc. Nephrol. 26 (2), 258–269. 10.1681/ASN.2014030278 25060060 PMC4310663

[B26] Kriz W. Lemley K. V. (2017). Mechanical challenges to the glomerular filtration barrier: adaptations and pathway to sclerosis. Pediatr. Nephrol. 32 (3), 405–417. 10.1007/s00467-016-3358-9 27008645

[B27] Lambert P. Aeikens B. Bohle A. Hanus F. Pegoff S. Van Damme M. (1982). A network model of glomerular function. Microvasc. Res. 23 (1), 99–128. 10.1016/0026-2862(82)90035-8 7099012

[B28] Layton A. T. Pham P. Ryu H. (2012). Signal transduction in a compliant short loop of Henle. Int. J. Numer. methods Biomed. Eng. 28 (3), 369–383. 10.1002/cnm.1475 PMC3346280 22577511

[B29] Layton H. Pitman E. B. Moore L. C. (1991). Bifurcation analysis of TGF-mediated oscillations in SNGFR. Am. J. Physiology-Renal Physiology 261 (5), F904–F919. 10.1152/ajprenal.1991.261.5.F904 1951721

[B30] Marsh D. J. Sosnovtseva O. V. Chon K. H. Holstein-Rathlou N. H. (2005a). Nonlinear interactions in renal blood flow regulation. Am. J. Physiology-Regulatory, Integr. Comp. Physiology 288 (5), R1143–R1159. 10.1152/ajpregu.00539.2004 15677526

[B31] Marsh D. J. Sosnovtseva O. V. Pavlov A. N. Yip K. P. Holstein-Rathlou N. H. (2005b). Frequency encoding in renal blood flow regulation. Am. J. Physiology-Regulatory, Integr. Comp. Physiology 288 (5), R1160–R1167. 10.1152/ajpregu.00540.2004 15661968

[B32] Mitrou N. Scully C. G. Braam B. Chon K. H. Cupples W. A. (2015). Laser speckle contrast imaging reveals large-scale synchronization of cortical autoregulation dynamics influenced by nitric oxide. Am. J. Physiology-Renal Physiology 308 (7), F661–F670. 10.1152/ajprenal.00022.2014 25587114

[B33] Navar L. Bell P. Evan A. (1986). “The regulation of glomerular filtration rate in mammalian kidneys,” in Physiology of membrane disorders. Editors Andreoli J. F. H. T. E. Fanestil D. Schultz S. G. (New York: Plenum), 637–667.

[B34] Navar L. G. Bell P. D. Burke T. J. (1982). Role of a macula densa feedback mechanism as a mediator of renal autoregulation. Kidney Int. Suppl. 12 (12), S157–S164. 6957671

[B35] Navar L. G. Arendshorst W. J. Pallone T. L. Inscho E. W. Imig J. D. Darwin Bell P. (2008). “Chapter 13 - the renal microcirculation,” in Microcirculation. Editors Tuma R. F. Durán W. N. Ley K. Second Edition (San Diego: Academic Press), 550–683.

[B36] Papenfuss H. D. Gross J. F. (1978). Analytic study of the influence of capillary pressure drop and permeability on glomerular ultrafiltration. Microvasc. Res. 16 (1), 59–72. 10.1016/0026-2862(78)90045-6 692459

[B37] Petermann A. T. Pippin J. Durvasula R. Pichler R. Hiromura K. Monkawa T. (2005). Mechanical stretch induces podocyte hypertrophy *in vitro* . Kidney Int. 67 (1), 157–166. 10.1111/j.1523-1755.2005.00066.x 15610239

[B38] Pries A. Secomb T. W. Gaehtgens P. (1996). Biophysical aspects of blood flow in the microvasculature. Cardiovasc. Res. 32 (4), 654–667. 10.1016/s0008-6363(96)00065-x 8915184

[B39] Pries A. Secomb T. W. Gessner T. Sperandio M. B. Gross J. F. Gaehtgens P. (1994). Resistance to blood flow in microvessels *in vivo* . Circulation Res. 75 (5), 904–915. 10.1161/01.res.75.5.904 7923637

[B40] Remuzzi A. Brenner B. M. Pata V. Tebaldi G. Mariano R. Belloro A. (1992). Three-dimensional reconstructed glomerular capillary network: blood flow distribution and local filtration. Am. J. Physiology-Renal Physiology 263 (3), F562–F572. 10.1152/ajprenal.1992.263.3.F562 1415586

[B41] Richfield O. Cortez R. Navar L. G. (2020). Simulations of glomerular shear and hoop stresses in diabetes, hypertension, and reduced renal mass using a network model of a rat glomerulus. Physiol. Rep. 8 (18), e14577. 10.14814/phy2.14577 32951361 PMC7507384

[B42] Richfield O. Cortez R. Navar L. G. (2021). Simulations of increased glomerular capillary wall strain in the 5/6-nephrectomized rat. Microcirculation 28, e12721. **n/a**(n/a). 10.1111/micc.12721 34192389 PMC9285434

[B43] Scully C. G. Mitrou N. Braam B. Cupples W. A. Chon K. H. (2016). Detecting interactions between the renal autoregulation mechanisms in time and space. IEEE Trans. Biomed. Eng. 64 (3), 690–698. 10.1109/TBME.2016.2569453 27244712

[B44] Scully C. G. Mitrou N. Braam B. Cupples W. A. Chon K. H. (2017). Detecting interactions between the renal autoregulation mechanisms in time and space. IEEE Trans. Biomed. Eng. 64 (3), 690–698. 10.1109/TBME.2016.2569453 27244712

[B45] Scully C. G. Siu K. L. Cupples W. A. Braam B. Chon K. H. (2013). Time–frequency approaches for the detection of interactions and temporal properties in renal autoregulation. Ann. Biomed. Eng. 41 (1), 172–184. 10.1007/s10439-012-0625-1 22810840

[B46] Sgouralis I. Layton A. T. (2012). Autoregulation and conduction of vasomotor responses in a mathematical model of the rat afferent arteriole. Am. J. Physiology-Renal Physiology 303 (2), F229–F239. 10.1152/ajprenal.00589.2011 PMC3404589 22496414

[B47] Sgouralis I. Layton A. T. (2014). Theoretical assessment of renal autoregulatory mechanisms. Am. J. Physiology-Renal Physiology 306 (11), F1357–F1371. 10.1152/ajprenal.00649.2013 PMC4042104 24623150

[B48] Sgouralis I. Layton A. T. (2015). Mathematical modeling of renal hemodynamics in physiology and pathophysiology. Math. Biosci. 264, 8–20. 10.1016/j.mbs.2015.02.016 25765886 PMC4426241

[B49] Sgouralis I. Maroulas V. Layton A. T. (2016). Transfer function analysis of dynamic blood flow control in the rat kidney. Bull. Math. Biol. 78 (5), 923–960. 10.1007/s11538-016-0168-y 27173401 PMC5902684

[B50] Shea S. M. (1979). Glomerular hemodynamics and vascular structure: the pattern and dimensions of a single rat glomerular capillary network reconstructed from ultrathin sections. Microvasc. Res. 18 (2), 129–143. 10.1016/0026-2862(79)90023-2 491981

[B51] Srivastava T. Thiagarajan G. Alon U. S. Sharma R. El-Meanawy A. McCarthy E. T. (2017). Role of biomechanical forces in hyperfiltration-mediated glomerular injury in congenital anomalies of the kidney and urinary tract. Nephrol. Dial. Transplant. 32 (5), 759–765. 10.1093/ndt/gfw430 28339567 PMC6075083

[B52] Takenaka T. Harrison-Bernard L. M. Inscho E. W. Carmines P. K. Navar L. G. (1994). Autoregulation of afferent arteriolar blood flow in juxtamedullary nephrons. Am. J. Physiology-Renal Physiology 267 (5), F879–F887. 10.1152/ajprenal.1994.267.5.F879 7977792

[B53] Walker M. (2001). “Dynamic modulatory interaction between myogenic and tubuloglomerular feedback mechanisms in the autoregulation of renal blood flow,” in Physiology (New Orleans, LA: Tulane University School of Medicine).

[B54] Walker M. Harrison-Bernard L. M. Cook A. K. Navar L. G. (2000). Dynamic interaction between myogenic and TGF mechanisms in afferent arteriolar blood flow autoregulation. Am. J. Physiology-Renal Physiology 279 (5), F858–F865. 10.1152/ajprenal.2000.279.5.F858 11053046

[B55] Weinstein A. M. (1986). A mathematical model of the rat proximal tubule. Am. J. Physiol. 250 (5 Pt 2), F860–F873. 10.1152/ajprenal.1986.250.5.F860 3706537

[B56] Zatz R. Dunn B. R. Meyer T. W. Anderson S. Rennke H. G. Brenner B. M. (1986). Prevention of diabetic glomerulopathy by pharmacological amelioration of glomerular capillary hypertension. J. Clin. investigation 77 (6), 1925–1930. 10.1172/JCI112521 PMC370553 3011862

